# Anti-inflammation effects of 8-oxo-9-octadecenoic acid isolated from *Undaria peterseniana* in lipopolysaccharide-stimulated macrophage cells

**DOI:** 10.17179/excli2018-1422

**Published:** 2018-08-02

**Authors:** Min-Cheol Kang, Young-Min Ham, Soo-Jin Heo, Seon-A Yoon, Su-Hyeon Cho, Seung-Hae Kwon, Myeong Seon Jeong, You-Jin Jeon, KKA Sanjeewa, Weon-Jong Yoon, Kil-Nam Kim

**Affiliations:** 1 Department of Marine Life Sciences, Jeju National University, Jeju 690-756, Republic of Korea; 2 Jeju Biodiversity Research Institute (JBRI), Jeju Technopark (JTP), Jeju 699-943, Republic of Korea; 3 Jeju International Marine Science Center for Research & Education, Korea Institute of Ocean Science & Technology (KIOST), Jeju 63349, Republic of Korea; 4 Chuncheon Center, Korea Basic Science Institute (KBSI), Chuncheon 200-701, Republic of Korea

**Keywords:** 8-Oxo-9-octadecenoic acid, anti-inflammatory, Undaria peterseniana, macrophages, lipopolysaccharide

## Abstract

The aim of this study was to investigate the anti-inflammatory activity of 8-oxo-9-octadecenoic acid (OOA) isolated from *Undaria peterseniana* by examining its ability to inhibit the lipopolysaccharide (LPS)-induced production of inflammatory mediators in RAW 264.7 macrophage cells. We found that OOA significantly suppressed the LPS-induced production of nitric oxide (NO) and inflammatory cytokines. OOA downregulated the LPS-induced expression of inducible nitric oxide synthase and cyclooxygenase-2 proteins. With respect to proinflammatory signaling pathways, OOA inhibited LPS-induced mitogen-activated protein kinase signaling by inhibiting the phosphorylation of c-Jun N-terminal kinase (JNK) and extracellular signal-regulated kinase (ERK). Moreover, OOA inhibited LPS-induced nuclear factor (NF)-κB signaling by reducing the phosphorylation of IκB-α and p50 proteins. These results indicate that OOA significantly reduces proinflammatory signaling, which results in reduced expression of cytokines and proinflammatory mediators. Taken together, these results suggest that OOA has potent anti-inflammatory effects and could be considered an effective anti-inflammatory agent.

## Introduction

Inflammation is a protective immune response of living tissue to injury such as damaged cells, irritants, and pathogens (Krautz et al., 2014[24]). However, an excessive inflammatory response can lead to various harmful effects in the human body, including cancer, cardiovascular disease, arthritis, and infectious disease (Lucas et al., 2006[27]). Macrophages play an important role in inflammatory responses by the immune system in various tissues of the body, and they can be activated by proinflammatory molecules, such as cytokines, chemokines, and lipopolysaccharide (LPS) (Duque and Descoteaux, 2014[13]; Newton and Dixit, 2012[32]). 

Among these inflammation-stimulating factors, LPS, an endotoxin, is commonly used to establish inflammatory models of macrophage cells and to assess the anti-inflammatory effects of natural substances (Huang et al., 2018[16]; Lin et al., 2017[25]). Previous studies have shown that stimulation of macrophages by LPS induces the synthesis of nitric oxide (NO) and increases the production of proinflammatory cytokines, including tumor necrosis factor-alpha (TNF-α) and interleukin 6 (IL-6) (Yang et al., 2012[41]; Chen et al., 2016[7]; Wang et al., 2016[38]). Many investigators have reported that multiple signaling pathways are activated in inflammatory responses in macrophages, such as those involving cyclooxygenase-2 (COX-2), inducible nitric oxide synthase (iNOS), and nuclear factor (NF)-κB (Chang et al., 2017[6]; Jeong et al., 2014[19]). By targeting these pathways, many researchers have tried to find anti-inflammatory agents that inhibit inflammatory mediator and cytokine production induced by LPS in macrophages (Tang et al., 2017[37]; Zar et al., 2014[42]). Fatty acids are carboxylic acids with long aliphatic chains. They have been shown to have biological activities, including antioxidant, anti-inflammatory, antibacterial, and anti-obesity effects (Fernando et al., 2016[14]; Korantzopoulos et al., 2005[23]). Among various biological materials from natural sources, fatty acids derived from seaweed have been reported to exhibit such biological activities (Mendes et al., 2013[30]; Airanthi et al., 2011[2]; Wan-Loy and Siew-Moi, 2016[39]). 

*Undaria peterseniana*, a type of edible brown seaweed, has been extensively used in foods in Korea and Japan. Several studies have reported that *U.* *peterseniana* has antioxidant, anti-inflammatory, and hair growth-promoting properties (Hwang et al., 2012[17]; Cho et al., 2013[10]; Kang et al., 2017[20]). However, the underlying mechanisms have not been clarified, and the compounds from *U.* *peterseniana* that are responsible for its anti-inflammatory effects have not been identified. Thus, the objective of this study was to measure the anti-inflammatory activity of 8-oxo-9-octadecenoic acid (OOA) isolated from *U.* *peterseniana *through its inhibition of cytokine production and inflammation-related signaling in macrophage cells.

## Materials and Methods

### Extraction and isolation of bioactive metabolites

**Figure 1:**
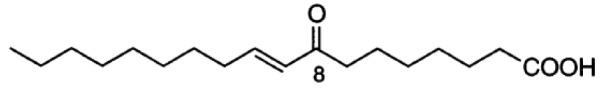
The chemical structure of 8-oxo-9-octadecenoic acid (OOA)

Seaweed (*Undaria peterseniana*) sample were supplied from the Jeju International Marine Science Research and Education Center of Korea Institute of Ocean Science and Technology (KIOST). Seaweed sample washed using running tap water (3 times) to remove the sand, salt, and epiphytes attached to the surfaces of *U. peterseniana*. The cleaned seaweeds were stored in a medical refrigerator at -20 °C until use. The frozen *U. peterseniana* was freeze-dried and homogenized using a grinder before starting the extraction procedure. The powdered *U. peterseniana* was extracted to 80 % methanol solution at room temperature and then methanol soluble part of seaweed concentrated using a vacuum evaporator. The 80 % methanolic extract was subsequently separated with chloroform using solvent-solvent partition chromatography. Then the chloroform soluble part further was purified using silica column chromatography with the stepwise elution of a mixture prepared from chloroform - methanol (100:1 - 1:1). The bio-active fraction was then feed to a Sephadex LH-20 column (GE Healthcare Bio-Sciences AB, Sweden) saturated with 100 % methanol, and finally purified using reversed-phase HPLC (ThermoFisher Scientific, Waltham, MA, USA) using a Waters HPLC system equipped with a Waters 996 photodiode array detector and a C18 column (J'sphere ODS-H80, 150 × 20 mm, 4 μm; YMC Co.) via stepwise elution with a methanol-water gradient (flow rate: 0.8 ml/min and UV range: 440 nm). The purity of isolated compounds was definitively identified through comparing their LC/MS, ^1^H, and ^13^C NMR data with those in the previous studies (Heo et al., 2008[15]). The purity of 8-Oxo-9-octadecenoic acid (OOA) (Figure 1(Fig. 1)) was higher than 95 %, based on the peak area of all components absorbed at each specific wavelength in HPLC analysis. OOA was dissolved in DMSO and used for cell culture studies in which the final concentration of DMSO in cell culture medium was adjusted to < 0.01 %.

### Cell culture and maintenance

The murine macrophage (RAW 264.7) cell line was purchased from the Korean Cell Line Bank (KCLB; Seoul, Korea). Macrophages were cultured in Dulbecco's modified Eagle's medium (DMEM). The DMEM was supplemented with 10 % fetal bovine serum (FBS), 100 μg/ml of streptomycin, and 100 U/ml of penicillin. DMEM, FBS and antibiotic were purchased from GIBCO, NY, USA. The cells were incubated at 37 °C with 5 % CO_2_. The macrophages were sub-cultured within two days' intervals.

### Cell viability assay

For determining the cytotoxicity of OOA, macrophages were cultured in 96-well plates at the density of 1.5 × 10^5^ cells/well, serum-starved for 24 h, and then treated with 1 μg/mL LPS and the selected concentrations of OOA for 18 h. Then, 50 µl of MTT solution (2 mg/ml) was added to each well, and RAW 264.7 macrophages were incubated for another 2 h at 37 °C. Then, the culture media and MTT discard, and DMSO was added to dissolve the purple precipitates. The absorbance of final solution was measured at 570 nm using an ELISA reader.

### Evaluation of NO inhibition

RAW 264.7 cells were cultured in 96 well plates with the density of 1.5 × 10^5^ cells/ml and incubated 24 h and then co-incubated with tested concentrations of samples and LPS. The quantity of nitrite accumulated in the cell free culture supernatants was measured using Griess assay. Briefly, 100 μl of culture supernatants was mixed with the equal volume of Griess reagent [0.1 % naphthylethylenediamine dihydrochloride in 2.5 % phosphoric acid and 1 % sulfanilamide]. Then the reaction mixture was incubated at room temperature for another 10 min and the absorbance was recorded at 540 nm using an ELISA microplate reader. The fresh culture medium was used as a blank throughout the study. The amount of nitrite in the culture supernatants was determined with a sodium nitrite standard curve.

### The protective effect of OOA against pro-inflammatory cytokines production (TNF-α and IL-6)

OOA was solubilized with DMSO and then diluted with DMEM before exposed to the cells. The inhibitory effect of 8-Oxo-9-octadecenoic acid on the production of pro-inflammatory cytokines (IL-6, and TNF-α) from LPS-exposed macrophages was according to the previously described method (Cho et al., 2000[9]). The cell free culture supernatants were used to evaluate suppressive effect of OOA against IL-6, and TNF-α secretion levels in LPS-stimulated macrophages. The ELISA assays were performed using mouse enzyme-linked immunosorbent assay (ELISA) kits following vendor's instructions (R&D Systems; Minneapolis, MN, USA).

### Western blot analysis

Murine macrophages were exposed to LPS (1 μg/ml) in the presence of OOA for the given times. After incubation, the macrophages were collected and washed twice using ice cold PBS to remove any remaining cell culture medium attached to the macrophages. Then the cells were lysed using a lysis buffer prepared by following a previously described method (Kim et al., 2010[21]). The cell lysates were separated using centrifuging at 15,00 RPM for 20 min. Then, the protein concentrations in the cell lysates were determined using a commercial protein quantification kit (BCA™ protein assay kit, ThermoFisher Scientific, Waltham, MA). Then, 30-50 μg of proteins obtained from cell lysis process were separated on 12 % SDS-polyacrylamide gel and transferred onto a polyvinylidene fluoride (PVDF) membrane with a glycine transfer buffer [25 mM Tris-HCl (pH 8.8), 192 mM glycine, and 20 % methanol (v/v)]. After blocking the nonspecific site of PVDF membranes with 5 % skim milk, the membranes were incubated overnight with target primary antibody at 4 °C in a cold room. The membranes were then incubated for another 1 h with a peroxidase-conjugated secondary antibodies separately (1:3000, Cell Signaling Technology, Beverly, MA, USA) at room temperature. The immune active proteins were visualized using an enhanced chemiluminescence (ECL) Western blotting detection kit (Amersham, Arlington Heights, IL, USA).

### Statistical analysis and data processing

All data are expressed as means ± S.D (n=3). Significant differences between the tested groups were determined using the unpaired Student's *t*-test and P<0.05 was considered as statistically significant differences.

## Results

### Inhibitory effect of OOA on LPS-induced NO generation 

**Figure 2:**
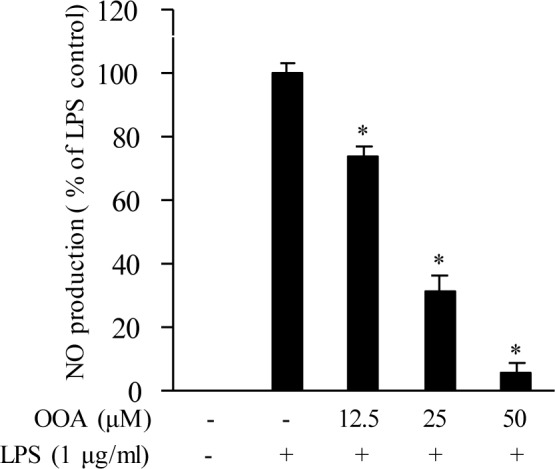
Effect of 8-oxo-9-octadecenoic acid (OOA) on production of NO in RAW 264.7 cells. Values are expressed as means ± S.D. of triplicate experiments. * *P *< 0.01 versus the LPS only group

An MTT (3-(4,5-dimethylthiazol-2-yl)-2,5-diphenyltetrazolium bromide) assay was used to determine the cytotoxicity of OOA against RAW 264.7 cells. OOA was not cytotoxic to RAW 264.7 cells at 12.5, 25, and 50 μM (data not shown). The inhibitory effect of OOA on NO production was measured using a Griess assay in LPS-stimulated RAW 264.7 cells. The results show that OOA significantly suppresses NO production in RAW 264.7 cells, indicating that OOA inhibits NO production in a concentration-dependent manner without cytotoxicity in macrophages (Figure 2(Fig. 2)).

### Inhibitory effect of OOA on the production of cytokines in LPS-stimulated RAW 264.7 cells

**Figure 3:**
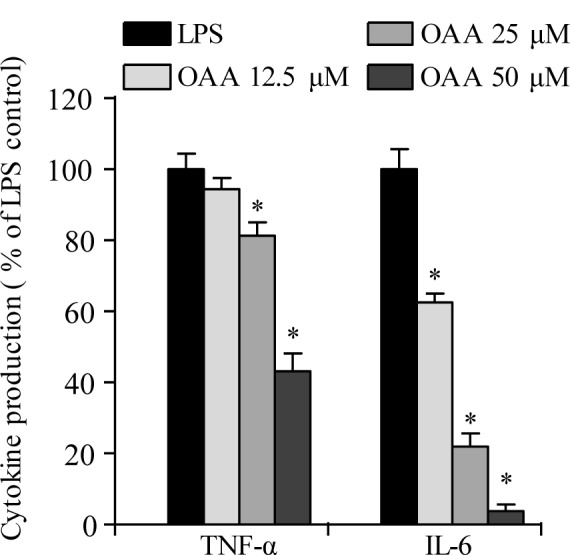
Effects of 8-oxo-9-octadecenoic acid (OOA) on TNF-α and IL-6 production in LPS-induced RAW 264.7 cells. Values are expressed as means ± S.D. of triplicate experiments. * *P *< 0.01 versus the LPS only group (black bars)

Cytokines, such as TNF-α and IL-6 are proinflammatory mediators in the pathogenesis of inflammation-related diseases. To examine the inhibitory effects of OOA on LPS-stimulated TNF-α and IL-6 production in RAW 264.7 cells, enzyme-linked immunosorbent assay (ELISA) kits were used to measure cytokine production. As shown in Figure 3(Fig. 3), OOA inhibited TNF-α and IL-6 production in a concentration-dependent manner in LPS-stimulated RAW 264.7 cells. These results demonstrate that OOA treatment inhibits NO production in LPS-stimulated RAW 264.7 cells, possibly by inhibiting TNF-α and IL-6 secretion. 

### OOA suppresses LPS-induced COX-2 and iNOS signaling pathways in LPS-stimulated RAW 264.7 cells 

**Figure 4:**
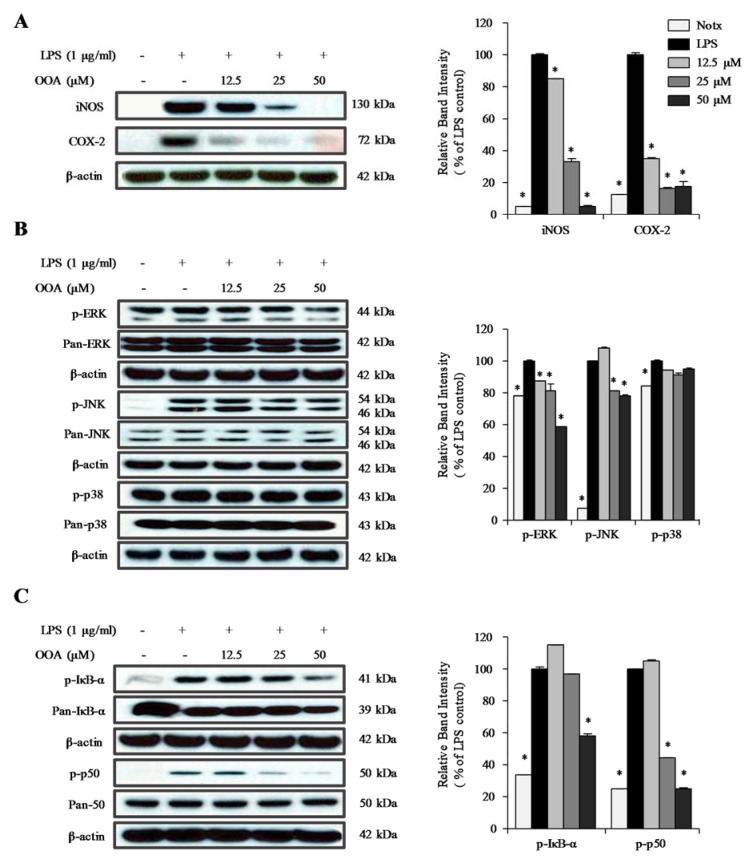
Inhibitory effect of 8-oxo-9-octadecenoic acid (OOA) on the protein levels of iNOS and COX-2 (A); phosphorylation of ERK, JNK, and p38 (B); and phosphorylation of IκB-α and p50 (C) in RAW 264.7 cells. Representative western blots are shown in the left panels and quantitative plots are shown to the right. Values are expressed as means ± S.D. of triplicate experiments. * *P *< 0.01 versus the LPS only group (black bars)

To further confirm the participation of COX-2 and iNOS in the OOA-mediated anti-inflammatory effects, the expression levels of COX-2 and iNOS were detected by immunoblotting in LPS-stimulated RAW 264.7 cells. The macrophage-like RAW 264.7 cells treated with LPS showed increased expression levels of COX-2 and iNOS (Figure 4A(Fig. 4)). When treated with OOA, the expression levels of COX-2 and iNOS were dramatically decreased compared with the levels in RAW 264.7 cells stimulated with LPS alone. These findings strongly indicate that OOA decreases NO production by inhibiting iNOS expression in RAW 264.7 cells. In addition, inflammatory prostaglandin synthesis may be decreased by the inhibition of COX-2 expression.

### Effects of OOA on the activation of mitogen-activated protein kinase (MAPK) signaling pathways 

MAPKs have been implicated in the regulation of proinflammatory cytokines and inflammatory signal transduction pathways. To elucidate the molecular mechanism of the OOA regulation of the LPS-induced MAPK pathway, immunoblotting analysis was conducted in RAW 264.7 cells. As shown in Figure 4B(Fig. 4), the phosphorylation of c-Jun N-terminal kinase (JNK) and extracellular signal-regulated kinase (ERK) was decreased in OAA- and LPS-treated cells compared with that of LPS-treated cells. However, treatment with OOA did not significantly affect the phosphorylation of p38. These results suggest that OOA inhibits inflammatory factors, including NO, TNF-α, and IL-6, by inhibiting both the phosphorylation of JNK and ERK in LPS-stimulated RAW 264.7 cells. 

### Inhibitory effect of OOA on LPS-induced phosphorylation of IκB-α and p50 protein

 Western blotting was performed to clarify the molecular mechanism of the anti-inflammatory effect of OOA; we analyzed the phosphorylation of IκB-α and p50 protein in RAW 264.7 cells. As shown in Figure 4C(Fig. 4), LPS-stimulation increased the phosphorylation of IκB-α and p50 protein in RAW 264.7 cells. Treatment with OOA significantly inhibited the phosphorylation of both IκB-α and p50 protein in a concentration-dependent manner. These results suggest that the inhibition of NO and cytokine production by OOA is associated with modulation of the NF-κB signaling pathway.

See also the Supplementary data.

## Discussion

The major objective of this study was to investigate the anti-inflammatory activities of OOA isolated from *U.* *peterseniana* by evaluating the inhibition of NO and inflammatory mediator production in macrophage cells. Among reactive oxygen species, excessively generated NO can attack many types of biological molecules (Abramson, 2008[1]). NO plays important roles in the pathogenesis of inflammation and the treatment of chronic inflammatory diseases can involve its targeting (Arulselvan et al., 2016[4]; Crosswhite and Sun, 2010[11]). We found that OOA has potent inhibitory effects on the production of NO in RAW 264.7 cells. In recent years, there has been increasing interest in cytokines as proteins that play critical roles in regulating both cellular and humoral chronic inflammatory responses (Nedoszytko et al., 2014[31]; Ko et al., 2016[22]). TNF-α and IL-6 are cytokines that are involved in systemic inflammation in the human body. TNF-α is a major proinflammatory cytokine and it can stimulate the production of IL-6 and IL-1β (Zhang and An, 2007[43]; Wojdasiewicz et al., 2014[40]). A number of investigators have reported that reducing macrophage cytokines plays a crucial role in anti-inflammatory effects (Scheller et al., 2011[35]; Ma et al., 2017[28]; Park et al., 2015[33]). Acute inflammatory activation of macrophages involves activation of MAPK- and NF-κB-mediated signaling pathways (Ivashkiv, 2011[18]; Chi et al., 2013[8]). De Souza et al. (2014[12]) reported that MAPKs are a group of serine/threonine protein kinases that regulate the expression of several cytokines, including TNF-α, IL-6, and prostaglandin E_2_. APK families are involved in cell proliferation, differentiation, survival, death, and apoptosis. Many studies have shown that LPS stimulation induces the activation of MAPKs including JNK, ERK, and p38 pathways. Especially, Ajizian *et al*. reported that JNK and ERK have a major involvement in the LPS-stimulated expression of iNOS and COX-2 in RAW 264.7 cells (Ajizian et al., 1999[3]; Bhat and Fan, 2002[5]). Previous studies have indicated that polyphenolic compounds isolated from seaweeds have anti-inflammatory effects by reducing cytokines through MAPK inhibition. These compounds include fucoxanthin isolated from brown algae, dieckol isolated from *Ecklonia cava*, and polyphenolics isolated from *Saccharina japonica*. In the same studies, fatty acids derived from the seaweeds were also shown to have anti-inflammatory effects by reducing cytokine production via inhibiting MAPK pathways in macrophages (Robertson et al., 2015[34]; McCauley et al., 2015[29]). In our present study, similar results were obtained, with OOA significantly inhibiting the LPS activation of ERK and JNK pathway, which suggests that OOA is at least partly responsible for the anti-inflammatory effects previously observed.

Furthermore, the NF-κB family of transcription factors plays a central role in the expression of proinflammatory mediators. The transcriptional activity of NF-κB is controlled to a large extent by the IκB family, which sequesters NF-κB in the cytoplasm. IκB-α is one family member that plays an important role in various inflammatory and pathological responses. IκB-α is an inhibitor of the nuclear translocation of NF-κB, but its expression also involves the MAPK signaling pathways, including JNK, ERK, and p38 pathways (Liu et al., 2017[26]; Tak and Firestein, 2001[36]; Zhang et al., 2005[44]). 

Based on these results, we suggest that OOA inhibits the production of NO, prostaglandins, and proinflammatory cytokines via the suppression of signaling pathways, which includes NF-κB activation and MAPK phosphorylation, in LPS-stimulated RAW 264.7 cells. Our results clearly indicate that OOA may be a potential therapeutic agent against inflammatory diseases. 

## Notes

Min-Cheol Kang and Young-Min Ham contributed equally as first authors.

Weon-Jong Yoon and Kil-Nam Kim (Chuncheon Center, Korea Basic Science Institute (KBSI), Chuncheon 200-701, Republic of Korea; Tel.: +82-33-815-4607, E-mail: knkim@kbsi.re.kr) contributed equally as corresponding authors.

## Acknowledgement

This research was supported by the Korea Basic Science Institute (C38260) and supported by the project of 'Development of integrated technologies for developing biomaterials using by magma seawater' in the 'Marine Biotechnology Program' funded by Ministry of Ocean and Fisheries, Korea and this research was supported by Basic Science Research Program through the National Research Foundation of Korea (NRF) funded by the Ministry of Education (NRF-2016R1D1A1B03933092).

## Conflict of interest statement

The authors declare that they have no conflict of interest.

## Supplementary Material

Supplementary data
